# Combined stress myocardial perfusion and late gadolinium enhancement imaging by cardiac magnetic resonance provides robust prognostic information to cardiac events

**DOI:** 10.1186/1532-429X-13-S1-O2

**Published:** 2011-02-02

**Authors:** Otavio Coelho-Filho, François-Pierre Mongeon, Kevin Steel, Ron Blankstein, Damien Mandry, Bobby Heydari, Michael Jerosch-Herold, Raymond Y Kwong

**Affiliations:** 1 Brigham and Women's, Boston, MA, USA

## Background

Accurate non-invasive risk stratification may management and impact survival of CAD patients. Stress perfusion CMR reliably assesses ventricular function, viability and myocardial ischemia in a single examination. While prognostic information may be derived from individual components of a comprehensive CMR exam, evidence that they provide complementary prognostic information is still limited. We sought to determine whether the presence of myocardial ischemia by stress perfusion CMR provides incremental prognostic information for major adverse cardiovascular events (MACE) beyond ventricular function, the presence of myocardial scar and traditional risk factors in a large cohort of patients referred for non-invasive assessment of CAD.

## Methods and results

**Figure 1:**
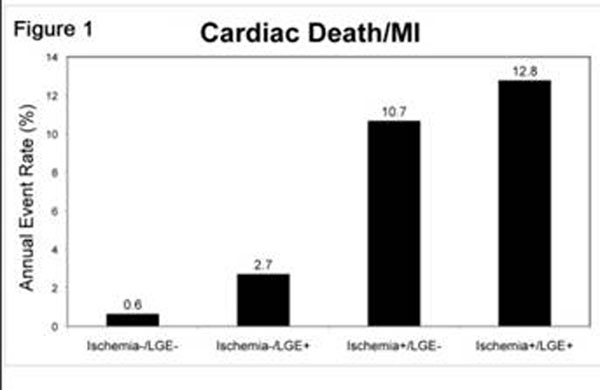
Cardiac Death/MI

**Figure 2:**
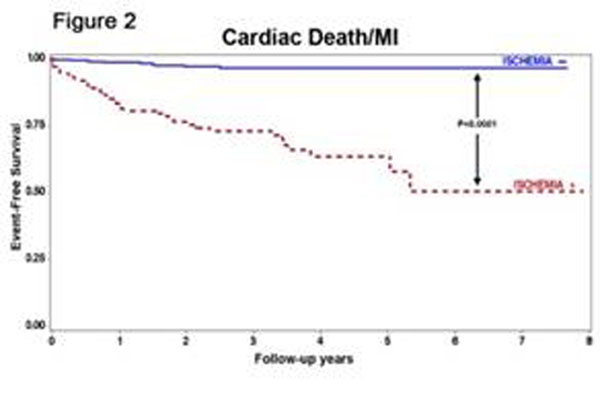
Cardiac Death/MI

Stress perfusion CMR was performed in 711 consecutive patients (297 females, mean age 56±15 years) referred to assess myocardial ischemia with an intermediate pre-test likelihood of CAD (mean pre-test likelihood of CAD 22±18%). Rest and vasodilator stress perfusion CMR were performed each using a 0.1mmol/Kg bolus infusion of gadolinium, followed by cine function imaging and late gadolinium enhancement (LGE) 10 minutes after a cumulative dose of 0.2mmol/Kg of gadolinium. The presence of myocardial ischemia was defined by a segmental stress-induced perfusion defect without matching segmental LGE. At a median follow-up of 21.4 months (range 2.5 months to 8.2 years), 52 MACE (8%) had occurred (29 cardiac deaths and 28 acute nonfatal MI). By univariable analysis, the presence of ischemia and LGE portended to > 11-fold and > 3-fold increases in MACE (LR*χ2,* 51.62 and 17.02, both P<0.0001, table1), respectively. Adjusting for age, LVEF, presence LGE and resting ST segment changes, presence of ischemia maintains a strong adjusted association with MACE (adjusted LR*χ2* 26.1, HR 7.4, P<0.0001). By stepwise forward selection (table 2) considering all pertinent clinical, CMR and ECG variables, presence of ischemia remained the strongest predictor of MACE in the best-overall model. A stress perfusion CMR study without ischemia and LGE predicted a very low negative annual MACE rate (0.6%, figure 1). In addition, the presence of ischemia was strongly associated with a reduced MACE-free survival (figure 2).

**Table 1:** Univariable prognostic association with MACE

Variable	LR_Χ_2	HR	P-Value
**Age, per decade**	21.12	1/06	<0.0001
**Gender**	0.05	0.94	0.8181
**Hypertension**	13.62	3.51	0.0002
**Diabetes**	13.17	2.80	0.0003
**Hyperlipidemia**	9.75	2.90	0.0018
**Hx MI**	10/80	2.58	0.0010
**Hx PCI**	11.60	2.71	0.0007
**HX CABG**	3.95	2.08	0.0469
**Pre-test Probability of CAD**	13.12	1.03	0.0003
**Left bundle branch block**	2.59	2.01	0.1074
**Significant Q Waves**	10.69	2.73	0.0011
**Resting ST changes**	27.43	4.48	<0.0001
**Resting T wave inversions**	10.73	2.57	0.0011
**LVEF, per 10%**	25.94	0.96	<0.0001
**LVEDVi, per 10 ml/m^2^**	13.54	1.01	0.0002
**LVESVi, per 10 ml/m^2^**	23.02	1.02	<0.0001
**Resting RWMA**	36.17	5.90	<0.0001
**Stress perfusion defect**	40.38	8.72	<0.0001
**Presence of LGE**	17.02	3.36	<0.0001
**ISCHEMIA presence**	51.62	11.53	<0.0001
**ISCH-SCORE**	84.06	1.19	<0.0001

**Table 2:** Best Overall model for MACE

Variable	LR_Χ_2	P-value	Hazard Ratio
**ISCHEMIA presence**	14.44	0.0001	5.038
**ISCH-Score**	7.60	0.0058	1.097
**Resting ST changes**	16.56	<0.0001	3.621

## Conclusion

The presence of ischemia by stress perfusion CMR provides robust prognostic information for MACE beyond the presence of scar, LVEF, and classical clinical and ECG markers of cardiac prognosis.. The combined absence of ischemia by myocardial perfusion imaging and scar by LGE imaging identifies a very low risk population.

